# Identification of the Non-Alcoholic Fatty Liver Disease Molecular Subtypes Associated With Clinical and Immunological Features *via* Bioinformatics Methods

**DOI:** 10.3389/fimmu.2022.857892

**Published:** 2022-07-25

**Authors:** Ziyu Liu, Yufei Li, Caihong Yu

**Affiliations:** School of Medicine, Hunan Normal University, Changsha, China

**Keywords:** molecular subtypes, bioinformatics, non-alcoholic fatty liver disease, inflammation, immune cell infiltration

## Abstract

**Background:**

Non-alcoholic fatty liver disease (NAFLD) is a manifestation of metabolic syndrome in the liver with varying severity. Heterogeneity in terms of molecules and immune cell infiltration drives NAFLD from one stage to the next. However, a precise molecular classification of NAFLD is still lacking, and the effects of complex clinical phenotypes on the efficacy of drugs are usually ignored.

**Methods:**

We introduced multiple omics data to differentiate NAFLD subtypes *via* consensus clustering, and a weighted gene co-expression network analysis was used to identify eight co-expression modules. Further, eigengenes of eight modules were analyzed with regard to Gene Ontology enrichment and Kyoto Encyclopedia of Genes and Genomes pathways. Furthermore, the infiltration rates of 22 immune cell types were calculated with CIBERSORT and the ESTIMATE algorithm.

**Results:**

In total, 111 NAFLD patients from three independent GEO datasets were divided into four molecular subtypes, and the corresponding clinical features and immune cell infiltration traits were determined. Based on high gene expression correlations, four molecular subtypes were further divided into eight co-expression modules. We also demonstrated a significant correlation between gene modules and clinical phenotypes. Moreover, we integrated phenotypic, immunologic, and genetic data to assess the potential for progression of different molecular subtypes. Furthermore, the efficacy of drugs against various NAFLD molecular subtypes was discussed to aid in individualized therapy.

**Conclusion:**

Overall, this study could provide new insights into the underlying pathogenesis of and drug targets for NAFLD.

## Introduction

With the growing incidence of non-alcoholic fatty liver disease (NAFLD), a corresponding increase in attention has been given to its classification in clinical practice. Traditionally, liver biopsy is considered the gold standard for the diagnosis and subtyping of NAFLD, but methodological challenges and evaluation uncertainties related to histological classifications could result in errors. Molecular subtypes are an effective supplement and an improvement to traditional histological classifications (1), and comprehensive considerations of histological patterns and molecular subtypes can be a realistic option for clinical assessments.

NAFLD is a manifestation of metabolic syndrome in the liver and a combination of hepatic and extrahepatic symptoms with different severity, but its complex clinical phenotypes with respect to the efficacy of drugs are usually ignored. Some clinical trials have not obtained satisfactory results. Some new drugs, for example, are effective for some phenotypes but ineffective or even harmful for others. The principal reason for this is the heterogeneity in NAFLD, and drug sensitivity among different subtypes has not been well differentiated. Further, current treatments for NAFLD are mainly based on lifestyle interventions, which is largely due to the lack of accurate studies from molecular and immunological perspectives. A new NAFLD classification should be precise in terms of dividing populations and defining the disease.

In summary, heterogeneity in terms of molecules and immune cell infiltration drives NAFLD from one stage to the next. This study focused on integrating phenotypes, immune cell infiltration, and genetic data to identify subpopulations with distinct pathogenic mechanisms and eigengenes. In addition, our work aimed to match specific treatment strategies with specific disease-driving factors and to evaluate the potential for progression of different NAFLD molecular subtypes to aid in individualized therapy.

## Materials and Methods

### Acquisition of NAFLD Datasets and Removal of Batch Effects

In total, datasets of 197 liver tissue samples (111 NAFLD, including 57 steatosis (SS) and 54 non-alcoholic steatohepatitis (NASH), as well as 86 healthy controls (HCs)) were collected from three Gene Expression Omnibus (GEO, https://www.ncbi.nlm.nih.gov/geo/) datasets (GSE48452 (2), GSE89632 (3), GSE151158 (4)) (5), which consisted of probe sets, clinical features, and gene expression profiles. The GSE48452 dataset (platform: GPL11532) consisted of 14 HC, 27 healthy obese, 14 SS, and 18 NASH cases. The GSE89632 dataset (platform: GPL14951) consisted of 24 HC, 20 SS, and 19 NASH cases. The GSE151158 dataset (platform: GPL28577) consisted of 21 HC, 23 SS, and 17 NASH cases. The datasets were integrated by surrogate variable analysis to reduce batch effects owing to the great batch differences between datasets from different platforms. The combat function was used to reduce the influence of known batch effects and other unknown variables (6), so that only the biological differences could be considered in the downstream analysis. Moreover, principal component analysis (PCA) was conducted to verify the results of the correction. The two factors that differed the most were selected as principal component 1 (PC1) and principal component 2 (PC2). Finally, the samples were mapped back to their own coordinates based on the selected PC1 and PC2 to obtain a dimensionality reduced image (PCA plot).

### Consensus Clustering of NAFLD Samples

The ConsensusClusterPlus R package was used to identify molecular subtypes of 111 NAFLD samples. Consensus clustering is a common method for the classification of cancer subtypes based on different omics datasets through a resampling method, specifying the number of clusters (k) and calculating the rationality of different numbers of clusters. For each cluster, the paired consensus values were calculated according to the occurrence rates of two samples in the same subsample of the same cluster, which were then stored in a consensus matrix. The consensus matrices were summarized in several graphs to help determine a reasonable number and the membership of clusters. Here, the K-means algorithm in the ConsensusClusterPlus R package was introduced to perform consensus clustering based on the Euclidean distance among NAFLD samples (7). The clustering processes were performed 500 times, with each iteration containing 80% of the samples. Finally, the consensus matrix, the consensus cumulative distribution function (CDF) plot, the delta area, the tracking plot, and the cluster-consensus plot were obtained. An optimal cluster number was determined based on these plots.

### Comparison of Clinical Characteristics Among Four Molecular Subtypes

A pairwise comparison based on arithmetic utilization was used to test the differences among the subgroups with regard to the clinical characteristics (**Supplemental Table 1**).

### Identification of Gene Co-Expression Patterns in Each Molecular Subtype

The differential gene expression analysis was performed among four NAFLD molecular subtypes and normal controls. For the identification of an upregulated differentially expressed gene (DEG), the average expression value of genes between two groups was set to be more than 0.2 with an adjusted p-value less than 0.05.

### Gene Set Enrichment Analysis

To sort DEGs in the four subgroups, gene set enrichment analysis (GSEA) was conducted by utilizing the predefined set of genes from those subgroups (8). Then, the predetermined set of genes was concentrated at the top or bottom of the list. When it appeared at the top of the list, the expression of this gene set was regarded as upregulated in general, whereas it was regarded as downregulated when it appeared at the bottom.

### Weighted Gene Co-Expression Network Analysis

Weighed gene co-expression network analysis (WCGNA) was performed on all NAFLD samples based on the DEGs in the four molecular subtypes (9) and the co-expression modules corresponding to the upregulated genes of each molecular subtype. Then, the relationships between the gene sets and sample phenotypes were analyzed, followed by the mapping of regulatory networks among genes in the gene set and the identification of key regulatory genes. Finally, a heat map was constructed using the genes of the eight co-expression modules (vertical axis) acquired from WGCNA in each molecular subtype and HC samples (horizontal axis).

### Analysis of the Correlation Between Module Characteristic Genes and Clinical Characteristics

The relationships among the characteristic genes of eight co-expressed modules, the NAFLD activity score, and the body mass index (BMI) in NAFLD patients were systematically analyzed.

### Gene Ontology Enrichment Analysis of Genes in Four Molecular Subtypes

After the acquisition of eight co-expression modules from WCGNA, eigengenes in the eight co-expression modules were analyzed for enrichment to visualize biological processes, and the most significantly enriched processes in each module were demonstrated.

### Functional Enrichment Analysis of Genes in Each Co-Expression Module

The MSigDB database (10) was used to obtain all gene sets related to the Kyoto Encyclopedia of Genes and Genomes (KEGG) pathways, and the eigengenes in the co-expression modules were enriched in relation to the KEGG pathway. The pathways showing the most significant enrichment in each module were selected for display.

### Immune Cell Infiltration in the Liver Tissues of NAFLD Patients

**Figure 1:**
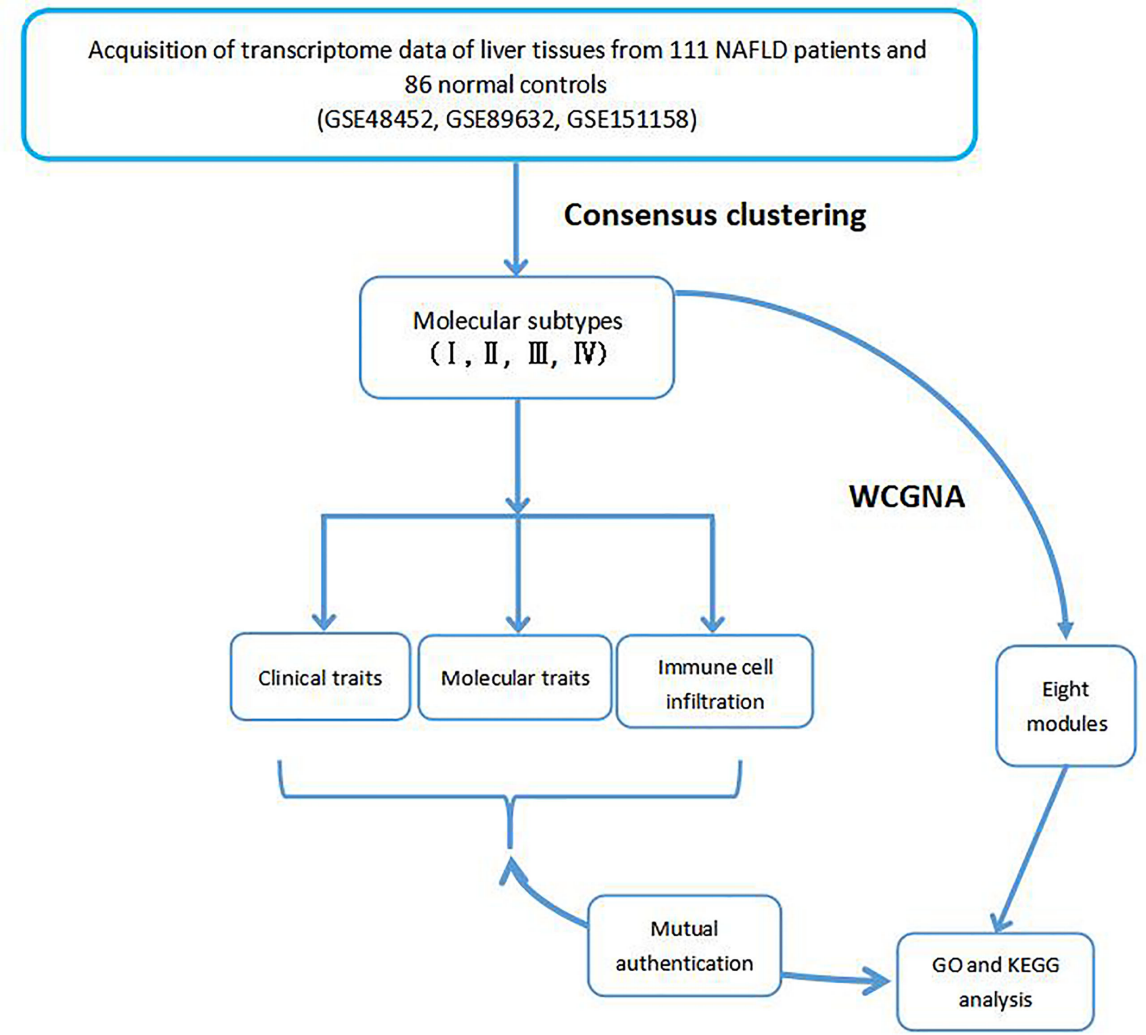
Flowchart of the integrated analysis.

The rates of infiltration of 22 types of immune cells in each NAFLD sample were calculated using the CIBERSORT algorithm and the expression data of 22 immune infiltrating cells, namely, the LM22 data (11). The ESTIMATE algorithm was employed to infer immune cell infiltration based on the transcriptome data and the scores of the immune microenvironment (12). These scores include the stromal score, the immune score, and the estimate score. In addition, the immune microenvironment scores and the corrplot R package were used to generate a correlation coefficient heatmap to visualize the prevalence of immune cell interactions in the immune microenvironment. The workflow of this study is shown in **Figure 1**.

## Results

### Collection of NAFLD Patient Clinical Information

In total, 197 samples were collected from three separate studies, including 57 SS, 54 NASH, and 86 HC cases. The clinical characteristics of each dataset are shown in **Supplemental Table 1**.

### Integration of Chip Data From Different Batches and Removal of Sample Batch Effects

**Figure 2:**
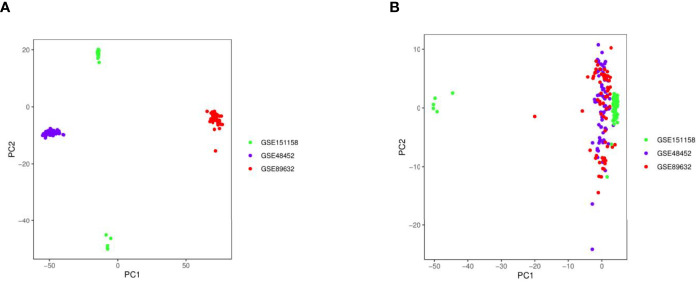
Principal component analysis (PCA) plots with or without the elimination of batch effects. **(A)** PCA plot without batch effect elimination; **(B)** PCA plot with batch effect elimination.

PCA plots with or without the elimination of batch effects are shown in **Figure 2**, in which the dots of different colors represent samples from different platforms. Samples were clearly divided into four groups. The results revealed that the influence of the data sources on the sample tests was greater than that on the sample types, indicating the presence of batch effects (**Figure 2** **A**). However, when the samples from three platforms were combined, the overall expression in the samples was distributed uniformly, indicating that the sample batch effects that influenced the calculation of molecular biological differences were eliminated (**Figure 2B**).

### Development of NAFLD Molecular Subtypes Using Consensus Clustering

**Figure 3:**
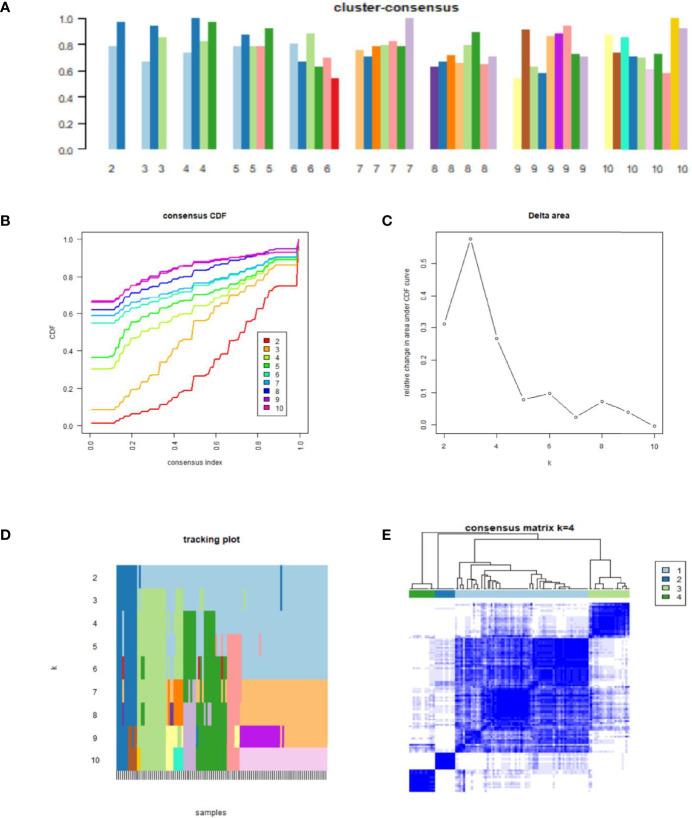
Results of consensus clustering analysis. **(A)** Cluster-consensus plot. Cluster-consensus plot showing the cluster-consensus value (the mean value of the pairwise consensus values of each subtype) with different k values. The higher the value, the more stable the subtype. **(B)** Cumulative distribution function (CDF) plot. The CDF plot shows the CDF of each cluster (K values corresponding to different clusters) for the selection of K, and the subtype is stable when the CDF descent has a small slope. **(C)** Delta area plot. The delta area plot shows the relative changes in the area under the CDF curve between K and K-1. When the area under the curve increases slightly, the K value is appropriate, and the classification is stable. **(D)** Tracking plot. The black stripes below the tracking plot represent the samples, which shows the classification of the samples at different k values, whereas the blocks of different colors represent different classifications. The classification is stable when colors of samples are scarcely changed at different K values. **(E)** Consensus matrix. The values of the consensus matrix are shown in white to dark blue from 0 (impossible to be clustered together) to 1 (always clustered together), and the consensus matrix was arranged according to the consensus clustering (dendrogram above the heatmap). The bars between the dendrogram and the heatmap represent the molecular subtypes. All results indicate that the sample clustering was stable and robust when the boundary of the consensus matrix was clear.

**Figure 3A** shows the clustering consistency scores of each subgroup with different cluster numbers. When the number of clusters was four, the clustering consistency scores of each subgroup were all greater than 0.7 (Figure 3A), and the consensus CDF plot (**Figure 3B**), delta area plot (**Figure 3C**), tracking plot (**Figure 3D**), and consensus matrix (**Figure 3E**) all showed stable results. Therefore, 111 total patients with NAFLD were divided into four clusters, namely, molecular subtype I (n = 67, including 38 NASH and 29 SS), molecular subtype II (n = 10, including 5 NASH and 5 SS), molecular subtype III (n = 21, including 8 NASH and 13 SS), and molecular subtype IV (n = 13, including 3 NASH and 10 SS).

### Analysis of Differences in Clinical Features Among Different NAFLD Molecular Subtypes

**Figure 4:**
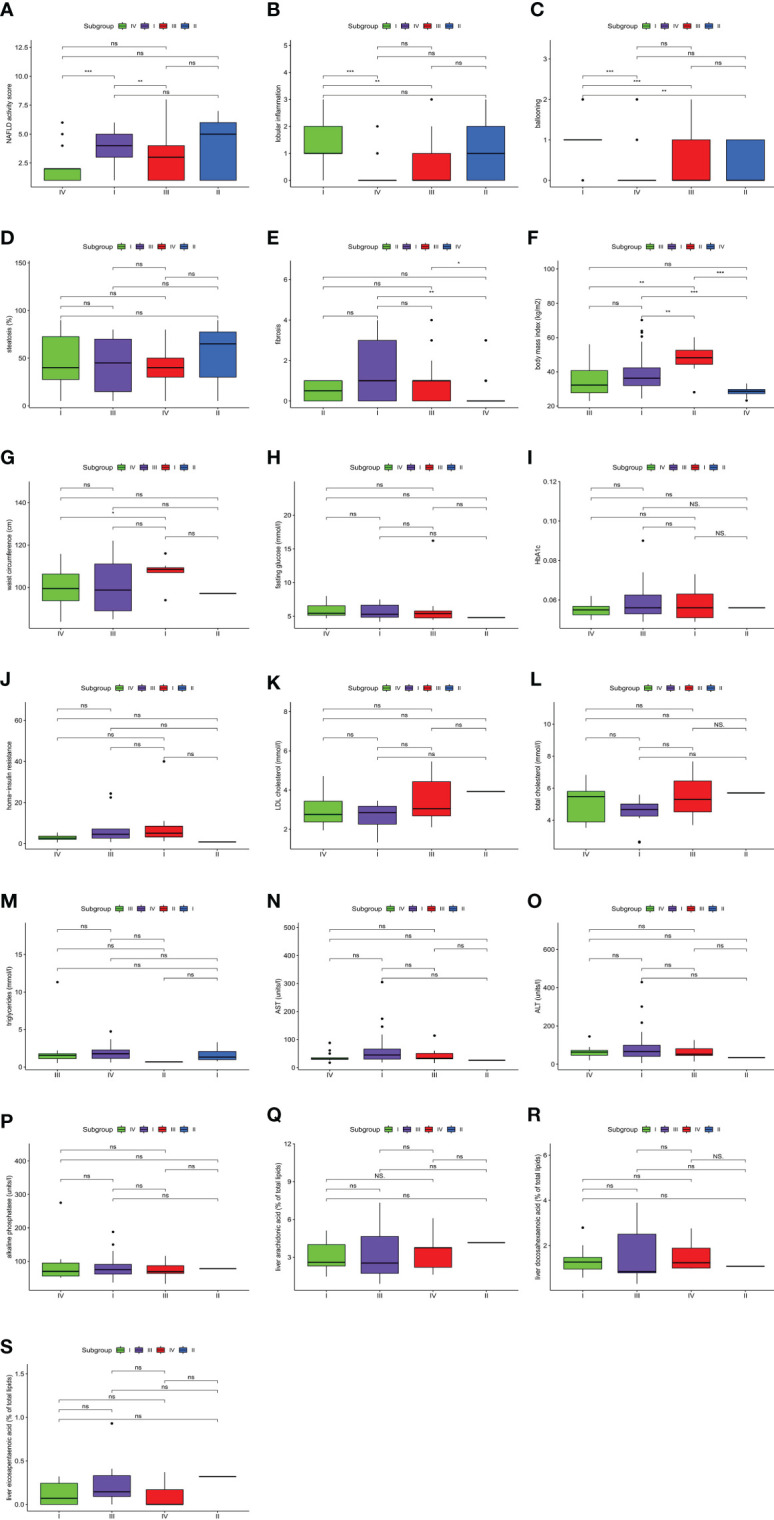
Clinical features of non-alcoholic fatty liver disease (NAFLD). The comparisons of clinical features among four molecular subtypes were conducted based on a pairwise Wilcoxon’s rank sum-test. **(A)** Difference in NAFLD activity score. **(B)** Difference in lobular inflammation. **(C)** Difference in ballooning. **(D)** Difference in steatosis. **(E)** Difference in fibrosis. **(F)** Difference in body mass index. **(G)** Difference in waist circumference. **(H)** Difference in fasting glucose. **(I)** Difference in HbA1c. **(J)** Difference in homa-insulin resistance. **(K)** Difference in LDL cholesterol. **(L)** Difference in total cholesterol. **(M)** Difference in triglycerides. **(N)** Difference in AST. **(O)** Difference in ALT. **(P)** Difference in alkaline phosphatase. **(Q)** Difference in liver arachidonic acid. **(R)** Difference in liver docosahexaenoic acid. **(S)** Difference in liver eicosapentaenoic acid. (*P < 0.05; **P < 0.01; ***P < 0.001; ns, not significant).

An analysis of clinical features among the four molecular subtypes was conducted (**Figure 4**). Lobular inflammation indicated the severity of the lesion, hepatocyte ballooning represented the intensity of the lesion, and fibrosis represented the stage of the lesion. According to the histological traits, molecular subtype I had the highest intensity and most advanced stage of the lesion. Molecular subtype II had the highest NAFLD activity score and the most fat deposition. The severity, intensity, and stage of molecular subtype III were mild. Molecular subtype IV had the lowest lesion intensity, the mildest severity, and the lowest lesion stage.

The NAFLD activity score and BMI were significantly different among molecular subtypes (P < 0.001). Furthermore, multiway ANOVA was used to compare the interaction between the two main effects, subgroups of the NAFLD activity score and BMI, as well as the interaction effect of the two variables on the NAFLD activity score (**Table 1**). This proved that the NAFLD activity score in this study was independent of BMI. These results not only suggested that the classification of patients based on gene expression profiles could distinguish between NAFLD activity scores but also demonstrated that BMI was an indicator of the severity of NAFLD. All of these results indicated the existence of significant heterogeneity among NAFLD patients.

**Table 1:** The statistical results of the two main effects based on the multiway ANOVA.

	Degree of freedom	Sum square	Mean square	F value	Pr (>F)
NAFLD activity score classification	3	36.92	12.308	4.702	0.00411
BMI	1	2.69	2.689	1.027	0.31323
NAFLD activity score classification and BMI	3	7.24	2.413	0.922	0.43325
Residuals	100	261.78	2.618		

NAFLD, non-alcoholic fatty liver disease; BMI, body mass index.

### Identification of Gene Co-Expression Patterns in Each Molecular Subtype

In the differential expression analysis among the four molecular subtypes, 46 DEGs were upregulated in subtype I, 130 DEGs were upregulated in subtype II, 52 DEGs were upregulated in subtype III, and 99 DEGs were upregulated in subtype IV. In addition, GSEA was conducted, and the results showed that those genes were significantly enriched in their respective molecular subtypes.

### WCGNA

**Figure 5:**
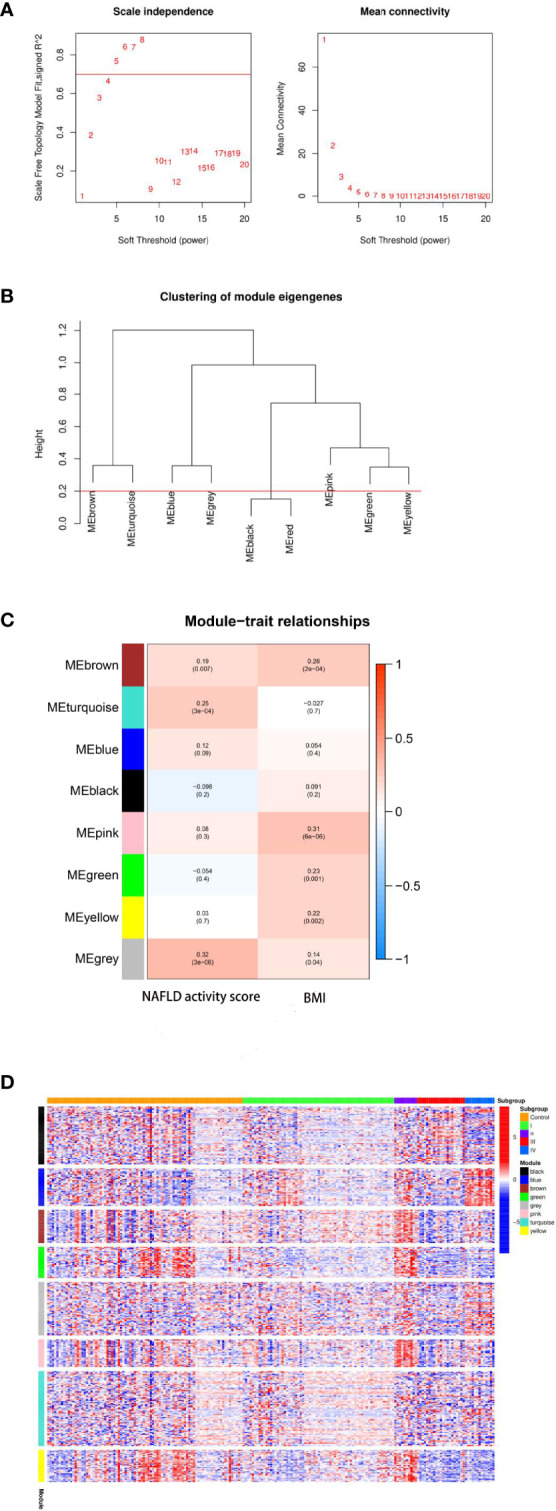
Results of weighted gene co-expression network analysis (WCGNA). **(A)** Calculation of the soft threshold. The absolute value of the correlation coefficient was set to Cex1 = 0.7, and the threshold was chosen to be 5. **(B)** Clustering of module eigengenes. The minimum height of the merged modules was set to 0.20, and eight total co-expression modules were obtained. **(C)** Relationships between modules and clinical traits. The gray module was the most correlated with the non-alcoholic fatty liver disease (NAFLD) activity score (r = 0.32, P = 3e-06), whereas the pink module was the most correlated with the body mass index (BMI; r = 0.31, P = 6e-06). **(D)** Heatmap of the co-expression modules corresponding to the upregulated eigengenes in each molecular subtype. Expression levels of genes (vertical axis of the figure) in eight co-expression modules and the control sample are shown on the horizontal axis of the figure as follows: transcriptome I corresponds to the turquoise module; transcriptome II corresponds to brown, green, and pink modules; transcriptome III corresponds to black and gray modules; transcriptome IV corresponds to the blue module.

WCGNA was performed based on 327 total genes that were differentially expressed among the four molecular subtypes of NAFLD as described. First, the soft threshold function of WGCNA was utilized to calculate the soft threshold, and a scale-free topological model was established (**Figure 5A**). Then, the dynamic hierarchical tree cutting algorithm was used to detect the co-expression modules (**Figure 5B**). The eight co-expression modules were compared with the two most varied clinical phenotypes among the four molecular subtypes, specifically the NAFLD activity score and BMI (**Figure 5C**). Gene modules that were highly correlated with the traits could be used for subsequent analysis to explore their biological functions. Finally, the expression patterns of specific upregulated genes from each molecular subtype were mapped using the pheatmap R package (**Figure 5D**).

### GO Enrichment Analysis of Genes Related to Molecular Typing and Co-Expression Modules in NAFLD

**Figure 6:**
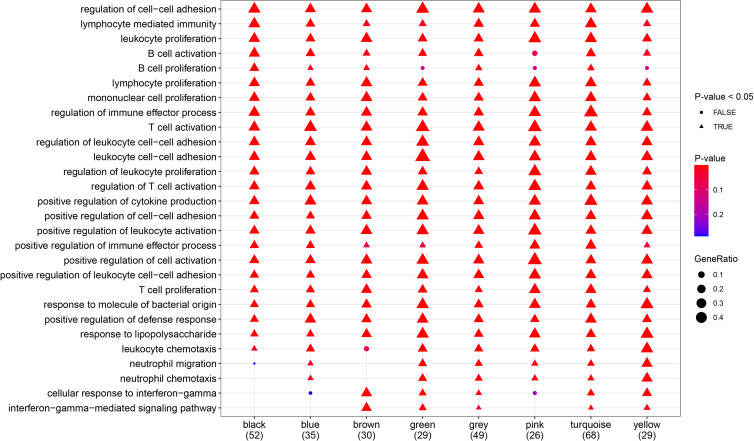
Gene Ont-ology (GO) analysis (biological processes). Most of the biological processes shown in the diagram were significantly enriched in all gene modules.

To further explore the relationships between gene modules and biological processes, protein–protein interaction (PPI) analysis and Gene Ontology (GO) enrichment analysis were performed using the STRING online tool (**Supplemental Table 2**). In addition, pathways for which all modules were enriched together were selected for display (**Figure 6**).

### Functional Enrichment Analysis of Genes in Each Co-Expression Module

**Figure 7:**
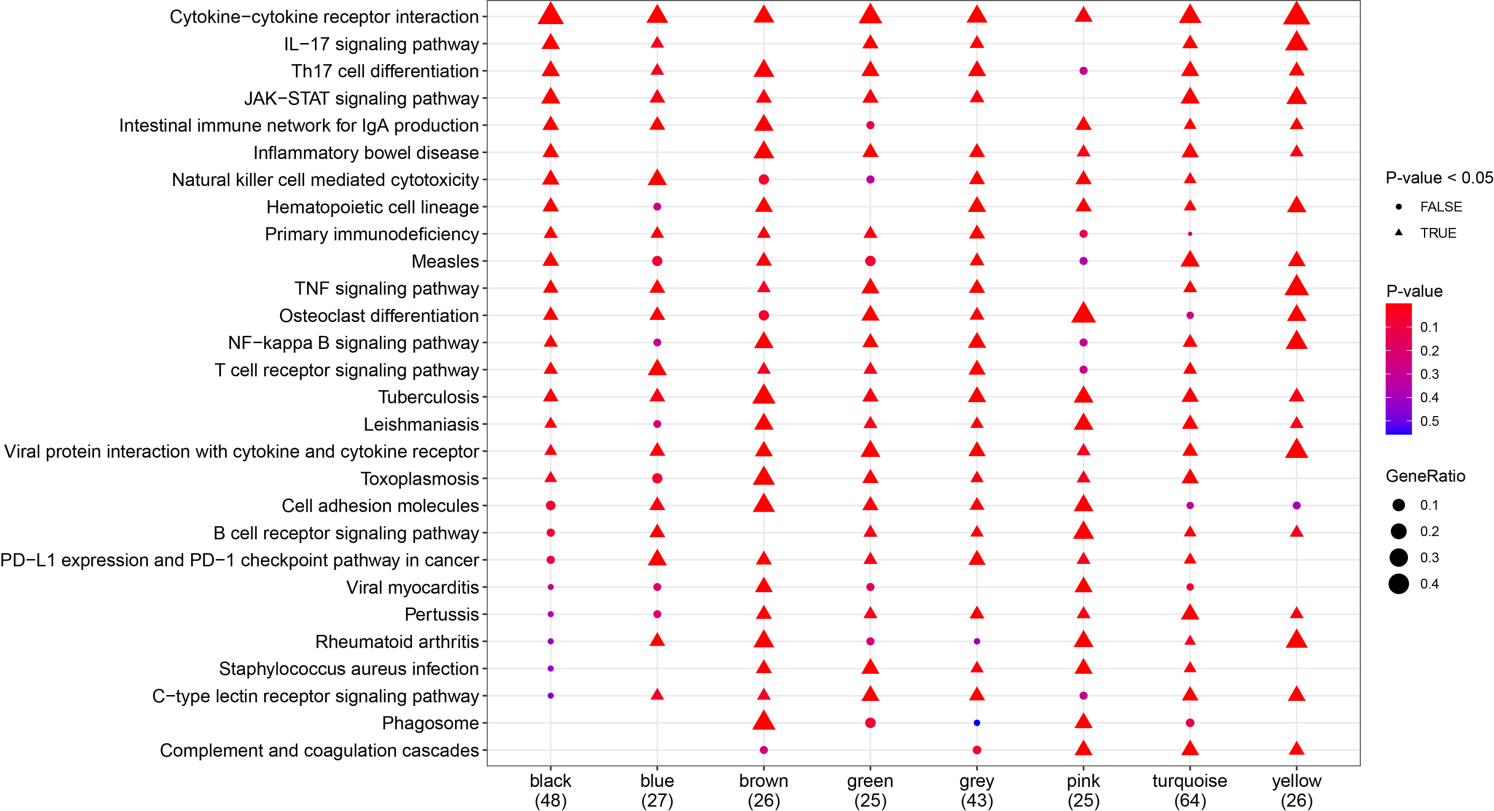
Kyoto Encyclopedia of Genes and Genomes (KEGG) pathway analysis. Demonstration of the pathways most significantly enriched. Triangles represent statistical significance (P < 0.05), and circles represent no statistical significance (P ≥ 0.05). The redder the color, the more significant the enrichment, and the larger the pattern, the more the genes are enriched. Different molecular subtypes were composed of different gene modules, which corresponded to different pathways.

To further explore the signaling pathways associated with the gene modules that regulate the clinical phenotypes, KEGG enrichment analysis was performed using the eigengenes in the co-expression modules, and the pathways that were most significantly enriched were selected for display (**Figure 7**). Pathways including cytokine–cytokine receptor interaction, Th17 cell differentiation, JAK-STAT signaling pathway, and inflammatory bowel disease were significantly enriched in more than six gene modules.

### Molecular Biological Characteristics of Four Molecular Subtypes

**Figure 8:**
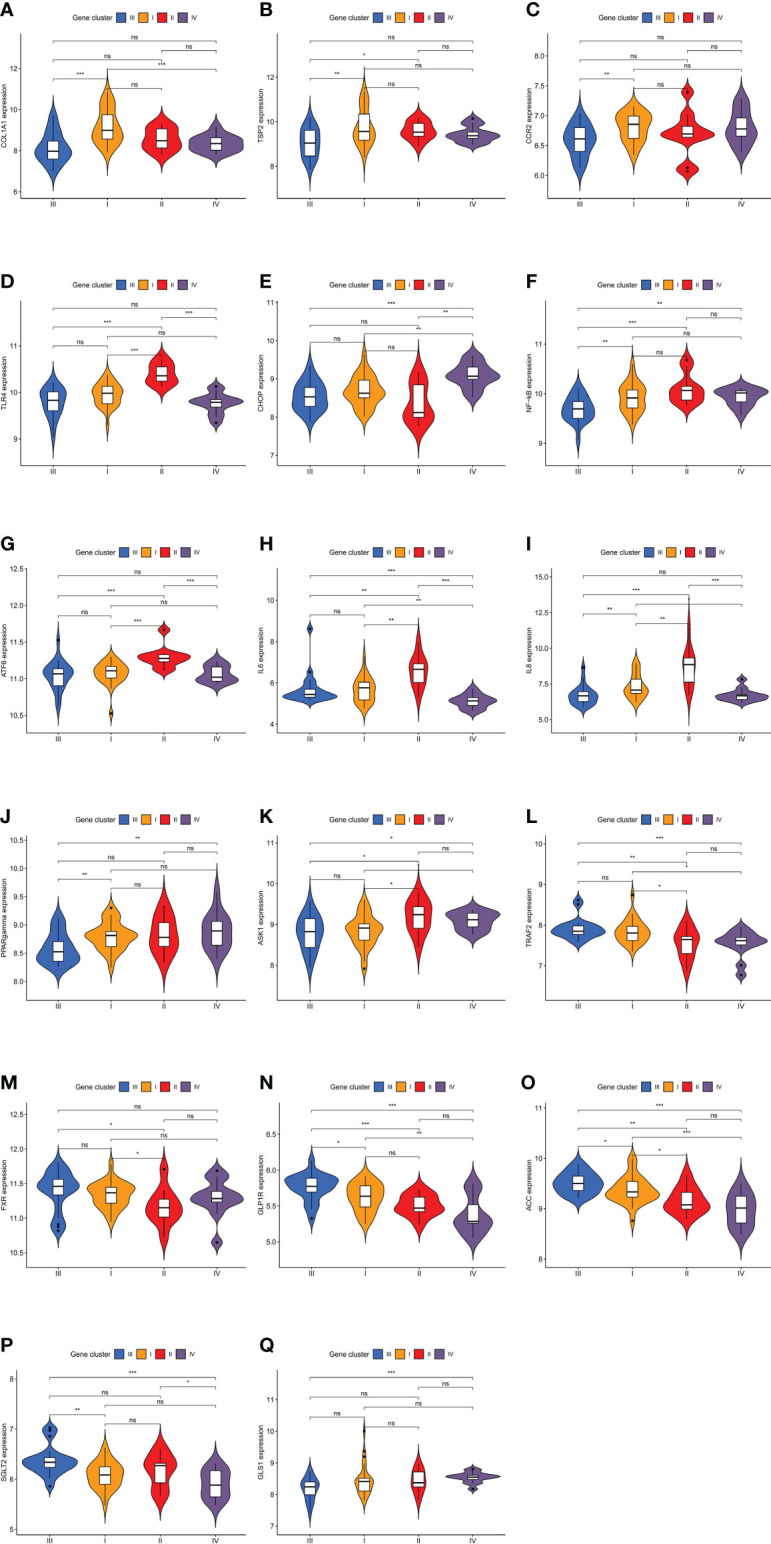
Expression levels of characteristic genes in four different molecular subtypes. The comparisons of genes among the four molecular subtypes were conducted based on the Kruskal–Wallis test. **(A)** Difference in COL1A1 expression level. **(B)** Difference in TSP2 expression level. **(C)** Difference in CCR2 expression level. **(D)** Difference in TLR4 expression level. **(E)** Difference in CHOP expression level. **(F)** Difference in NF-kB expression level. **(G)** Difference in ATF6 expression level. **(H)** Difference in IL6 expression level. **(I)** Difference in IL8 expression level. **(J)** Difference in PPARgamma expression level. **(K)** Difference in ASK1 expression level. **(L)** Difference in TRAF2 expression level. **(M)** Difference in FXR expression level. **(N)** Difference in GLP1R expression level. **(O)** Difference in ACC expression level. **(P)** Difference in SGLT2 expression level. **(Q)** Difference in GLS1 expression level. (*P < 0.05; **P < 0.01; ***P < 0.001; ns, not significant).

The Kruskal–Wallis test was used to compare the expression levels of characteristic genes among the four different molecular subtypes (**Figure 8**). The associations between the expression levels of those genes and clinical traits are presented in the discussion section.

### Immune Cell Infiltration in the Liver Tissues of Patients With NAFLD

**Figure 9:**
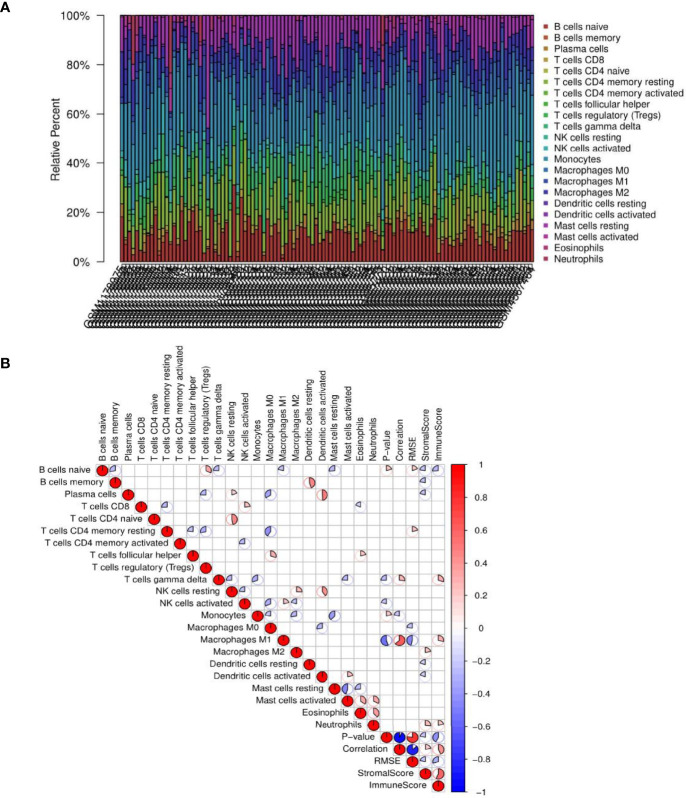
Results of immune cell infiltration. **(A)** Relative percentages of immune cells in each non-alcoholic fatty liver disease (NAFLD) sample. **(B)** Correlation coefficient heatmap for the visualization of the prevalence of immune cell interactions in the immune microenvironment of NAFLD patient liver tissues. The heatmap shows the associations among 22 immune cell populations. The color red represents positive associations, and the color blue represents negative associations. The size of the sector area of each grid represents the size of the correlation coefficient. The darker and more significant the grid, the stronger the correlation between the corresponding horizontal and vertical objects of the grid. Blanks in the graph indicate that the P-value had not reached the threshold, and the color is marked only when the P-value was less than 0.05.

The results showed that the panorama of immune cells varied greatly among patients (**Figure 9A**). Further, a correlation coefficient heatmap was prepared to visualize the associations among 22 immune cells in the immune microenvironment (**Figure 9B**).

### Comparisons of Immune Cell Subsets Among NAFLD Molecular Subtypes

**Figure 10:**
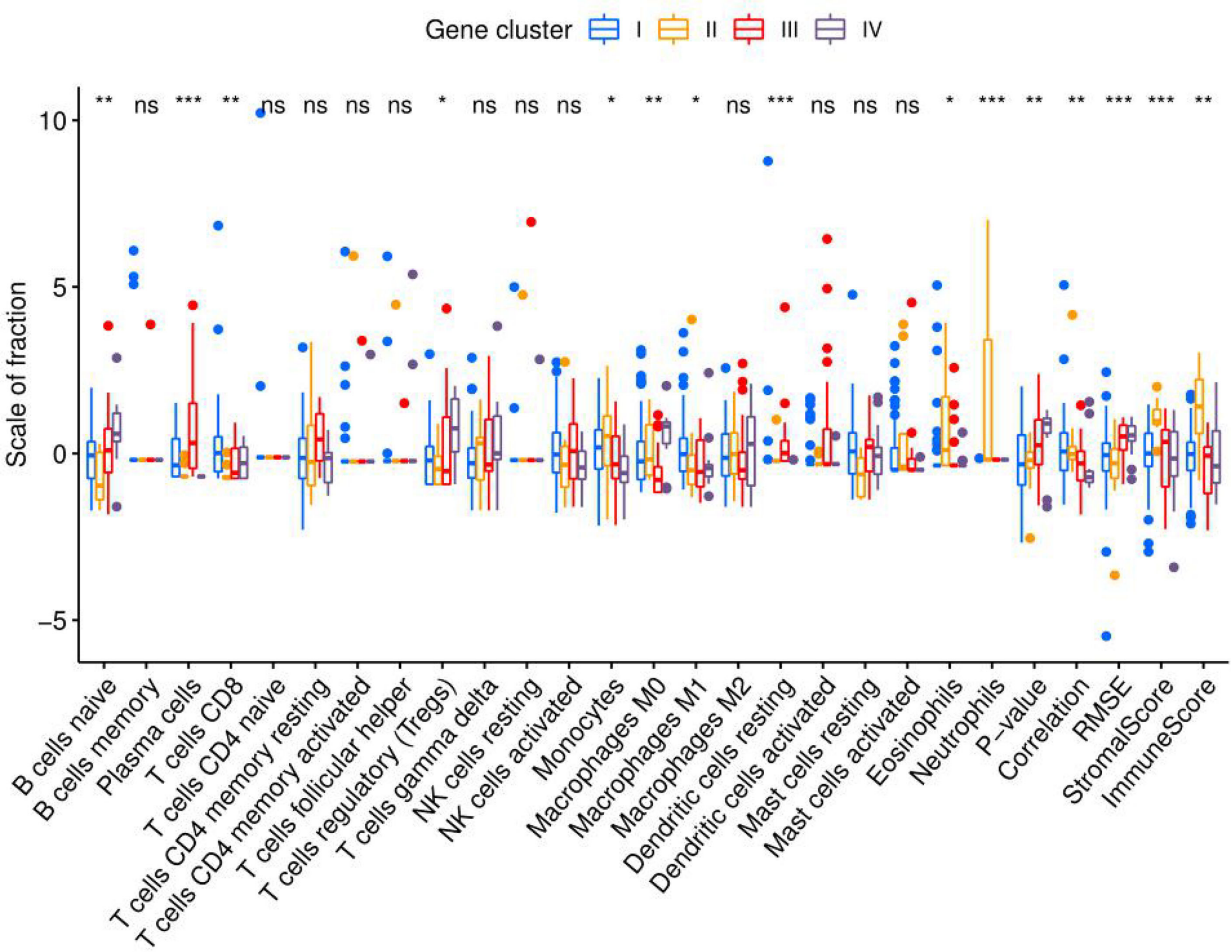
Immune cell infiltration in molecular subtypes I, II, III, and IV. Eosinophils, neutrophils, and monocytes of molecular subtype II were significantly more abundant than those in the other molecular subtypes. The ratio of M1 macrophages to M2 macrophages was ranked as follows: subtypes IV < II < III < I. (*P < 0.05; **P < 0.01; ***P < 0.001; ns, not significant).

To compare the differences in the distribution among the four molecular subtypes, the genotyping results and immune microenvironment scores were applied. The CIBERSORT algorithm was used to evaluate the 22 immune cell populations in each molecular subtype with a threshold of P < 0.05 (**Figure 10**). According to the immune score, the severity of the inflammatory response from mild to severe was ranked as follows: subtype IV < III < I < II. Through the analysis of immune cell infiltration, the immune characteristics of each molecular subtype were determined.

## Discussion

Based on gene expression profiles, 111 NAFLD patients from three independent GEO datasets were divided into four molecular subtypes, and the corresponding clinical features and immune cell infiltration traits were determined. In addition, based on the good correlation for gene expression results, four molecular subtypes were further subdivided into eight co-expression modules. The modules belonging to the same molecular subtype had similar clinical phenotypes and immunological characteristics. In addition, the significant correlations between gene modules and clinical phenotypes were also demonstrated. Furthermore, phenotypic, immunological, and genetic data were integrated to assess the potential for progression among the different molecular subtypes. In addition, the efficacy of drugs against different molecular subtypes of NAFLD was analyzed to aid in individualized therapy.

### Characteristics of Molecular Subtype I NAFLD

The histological features of molecular subtype I were significant fibrosis and a wide range of hepatocyte ballooning, with the greatest intensity and highest stage of the lesion. Molecular subtype I was clinically characterized by high levels of ALT, AST, and ALP, indicating that the degree of liver cell injury associated with molecular subtype I was serious, and its lesions influencing the hepatic parenchyma were the largest among the four molecular subtypes, which is consistent with the results of the histological analysis. The infiltration of M1 macrophages and CD8 T cells in molecular subtype I was significantly greater than that in the other molecular subtypes. The cytokine network produced by CD8+ lymphocytes is a potent activator of M1 macrophages. Oxidative damage *via* activated M1 macrophages accelerates the progression of NASH fibrosis (13). GO analysis showed that the turquoise module belonging to molecular subtype I was involved in the positive regulation of amyloid fibril formation, a biological process closely related to fibrosis. In conclusion, the histological characteristics (significant fibrosis), enrichment analysis, and immune cell infiltration results (marked infiltration of M1 macrophages and CD8 T cells) all indicated that molecular subtype I was associated with significant fibrosis, and thus, molecular subtype I was named fibrotic NAFLD. Further, collagen type I alpha 1 chain (COL1A1) (14) and thrombospondin 2 (TSP2) (15) exhibited the highest expression levels in molecular subtype I as biomarkers of the advanced fibrosis stage of NAFLD (**Figures 8A, B** **)**, demonstrating that molecular subtype I had the most significant fibrosis. The treatment of molecular subtype I NAFLD should focus on decreasing liver collagen deposition and increasing fibrinolysis.

Inflammation is an important factor driving liver fibrosis, and anti-inflammatory approaches and oxidative stress suppression are ideal methods to relieve liver fibrosis. C–C motif chemokine ligand 2 (CCL2) can recruit monocytes for infiltration and further promote inflammatory mediator production and oxidative damage by inducing differentiation into activated M1 macrophages (16). Clinical trials on cenicriviroc, a CC-chemokine receptor 2 (CCR2) antagonist, as a means to block CCL2 signaling, have shown significant improvements in relieving fibrosis progression with NASH (17). CCR2 expression was most significantly increased in molecular subtype I compared with that in other molecular subtypes (**Figure 8C**), indicating that cenicriviroc could have better fibrosis-attenuating effects on molecular subtype I and would be therefore more suitable for patients with this molecular subtype.

### Characteristics of Molecular Subtype II NAFLD

The histological characteristics of molecular subtype II included a high NAFLD activity score and large amounts of steatosis. Patients with molecular subtype II had a high BMI as the main clinical feature, and it was the only molecular subtype with a mean BMI greater than 40 kg/m². It was suggested that weight loss measures are particularly important for this molecular subtype. The immune score of molecular subtype II was significantly higher than that of the other molecular subtypes, indicating that the inflammatory response in molecular subtype II was the most intense. The microflora promotes NAFLD through γδ T cells, which are a subgroup with pro-inflammatory and anti-infection functions (18). γδ T cells and monocytes were significantly more abundant in molecular subtype II. Consistently, GO analysis showed that the brown module of molecular subtype II involved several biological processes closely related to microbial infection, such as detection of lipopolysaccharide (LPS) and peptide antigen assembly with major histocompatibility complex class II protein. In addition, elevated bacterial LPS levels could activate Toll-like receptor 4 (TLR4) and lead to NAFLD (19). Therefore, we examined TLR4 expression and found that it was significantly higher in molecular subtype II than in the other molecular subtypes (**Figure 8D** **)**. In summary, the results of immune cell infiltration, gene enrichment, and molecular expression data all suggested that molecular subtype II was closely associated with microbial infection. Microbial infection is an underlying cause of NAFLD, and treatment options based on targeting the microbiota comprise a recent hot topic in this field (20). Our study showed that microbiologically induced NAFLD belongs to molecular subtype II.

DNA damage-inducible transcript 3 (CHOP), a major gene involved in the NAFLD stress response, can trigger inflammatory responses by activating nuclear factor-kB (NF-kB) signaling in saturated fat cells (21). The expression levels of CHOP and NF-kB, two major genes involved in the stress response, were significantly higher in molecular subtype II than in the other three molecular subtypes (**Figures 8E, F**). Activating transcription factor 6 (ATF6), as one of the three sensor proteins of the unfolded protein response (a key link between cell stress and inflammation), is an important regulator of the endoplasmic reticulum stress pathway (22), and ATF6 expression was significantly higher in molecular subtype II than in the other molecular subtypes (**Figure 8G**). The high expression of a series of genes promoting the stress response indicated that the stress response in molecular subtype II was more intense than that in the other molecular subtypes. Moreover, a significantly high expression of some genes closely related to inflammatory responses (IL-6 and IL8) was discovered in molecular subtype II (**Figure 8H, I**), indicating that its inflammatory response was more intense than that of the other molecular subtypes. Fat accumulation could promote inflammation and the stress response (19), and those processes, found to have a significant role in molecular subtype II, are continuous and closely related. In conclusion, molecular subtype II was named the fat accumulation–inflammation–stress type of NAFLD. The treatment of molecular subtype II should thus focus more on improving insulin sensitivity, inhibiting fat deposition and inflammatory cell aggregation, blocking inflammatory signal transduction, and reducing the stress response.

Selonsertib is a potent and selective apoptotic signaling regulated kinase-1 (ASK1) inhibitor that ameliorates liver steatosis and inflammation (23). ASK1 expression was significantly higher in molecular subtype II than in the other molecular subtypes (**Figure 8K**), suggesting that selonsertib could achieve better therapeutic efficacy for patients with molecular subtype II. Further, the TLR4 antagonist JKB-121 is a long-acting small molecule and an effective treatment for the prevention of inflammatory injury and liver fibrosis in NAFLD patients (23). According to the high expression of TLR4 in molecular subtype II (**Figure 8D**), JKB-121 appears to be more effective for patients with this subtype.

### Characteristics of Molecular Subtype III NAFLD

The severity, intensity, and stage of molecular subtype III were mild. Its histological features were minor fibrosis, a small range of steatosis, and mild lobular inflammation. Clinical indexes, including AST, ALT, and ALP, were lower than those in molecular subtype I, suggesting that liver damage in molecular subtype III was less severe than that with subtype I. In molecular subtype III, plasma cells infiltrated redundantly. Under the stimulation of oxidative stress, liver B2 lymphocytes mature into plasma cells (24) that produce oxidative stress epitope (OSE) IgGs (13), resulting in the onset of NAFLD. As an early event in the evolution of NAFLD (24), the infiltration of plasma cells could help to identify molecular subtype III, which was named plasmacyte-infiltrating NAFLD according to its immune cell infiltration traits. In addition, the expression of TNF receptor-associated factor 2 (TRAF2), the central regulator of the cell response to stress (25), was significantly higher in molecular subtype III than in the other molecular subtypes (**Figure 8L**). In terms of cell stress, this is consistent with the immune cell infiltration results showing that plasma cell infiltration was abundant in molecular subtype III, which verifies the validity of our conclusion from a different perspective. Th2 cells have a high potential for promoting fibrosis *via* IL-13 (26). The production of cytokines by Th2 cells, as well as the production and secretion of IL-13, was negatively regulated in the black module of molecular subtype III (**Supplemental Table 2**), indicating that the progression of fibrosis in the early stage of NAFLD is inhibited.

Obeticholic acid is a potent farnitol X receptor (FXR) agonist that reduces liver enzymes and improves the degree of liver fibrosis. FXR expression was significantly higher in the molecular subtype III than in the other molecular subtypes (**Figure 8M**), indicating that patients with molecular subtype III would be more sensitive to FXR and that obeticholic acid is more suitable for these patients. Liraglutide is a glucagon-like peptide 1 receptor (GLP-1R) agonist that can effectively control blood glucose and improve metabolism, and it has a significant effect on reducing liver inflammation, steatosis, and fibrosis. The expression of GLP-1R in molecular subtype III was significantly higher than that in the other molecular subtypes (**Figure 8N**), indicating that liraglutide would be more suitable for patients with molecular subtype III. GS-0976, an inhibitor of acetyl-CoA carboxylase (ACC), can significantly reduce liver fat content and TIMP-1 (a serum marker associated with fibrosis) (23). ACC expression was significantly higher in molecular subtype III (**Figure 8O**), indicating that GS-0976 would be more suitable for patients with this subtype. Sodium-dependent glucose transporter 2 (SGLT2) inhibitors (canagliflozin (27), luseogliflozin (28)) are promising drugs that can reduce ALT levels and alleviate hepatic steatosis and fibrosis (29). SGLT2 expression was significantly higher in molecular subtype III than in the other molecular subtypes (**Figure 8P**), suggesting that SGLT2 inhibitors are more suitable for patients with molecular subtype III.

### Characteristics of Molecular Subtype IV NAFLD

Molecular subtype IV had the smallest lesion intensity, the mildest severity, and the lowest lesion stage. Except for a small range of steatosis, other indicators of NAFLD grading were low. According to its histological characteristics, molecular subtype IV was named primary NAFLD. Levels of clinical indictors, including ALP, BMI, HbA1c, and low-density lipoprotein cholesterol, were all lower in molecular subtype IV than in the other molecular subtypes, indicating that molecular subtype IV had the mildest clinical manifestation. Further, molecular subtype IV had the lowest immune score. The ratio of M1 to M2 macrophages was also lowest among all molecular subtypes, which indicated that molecular subtype IV exhibits the mildest inflammatory response. The gray module of molecular subtype IV was involved in the apoptotic process of B cells, as well as the negative regulation of T-cell cytokine production (**Supplemental Table 2**). This suggested that immunity in molecular subtype IV is negatively regulated, which verifies the results of immune cell infiltration from the perspective of gene enrichment.

Pioglitazone is an insulin sensitizer that acts on the peroxisome proliferator-activated receptor γ (PPARγ) to reduce steatosis by increasing lipid oxidation, thereby preventing NASH (23). PPARγ expression in molecular subtype IV was found to be significantly higher relative to that in the other molecular subtypes (**Figure 8J**), indicating that this subtype is the most responsive to pioglitazone. Inhibitors of hepatic glutaminase 1 (GLS1) reduce NASH steatosis by increasing very low-density lipoprotein export, and inhibiting GLS1 can reduce oxidative stress in NASH. GLS1 hepatic targeting has been shown to be a valuable therapeutic method (30), and GLS1 expression was higher in molecular subtype IV than in the other molecular subtypes (**Figure 8Q**). This suggested that this novel therapeutic method for NAFLD might be more suitable for patients with molecular subtype IV.

### Analysis of NAFLD Clinical Indicators Among the Four Molecular Subtypes

Combined with the characteristics of each molecular subtype analyzed previously herein, four clinical indicators were used to verify the reliability of typing. BMI showed a decreasing trend among subtypes in the following order: II, I, III, IV (**Figure 4F**). Meanwhile, triglycerides (TGs) showed an increasing trend among subtypes in the following order: II, I, III, IV (**Figure 4M**). The changing trends in BMI and TGs were opposite, and thus, we speculate that TG, BMI, and NAFLD might be related. Studies have shown that the effect of BMI on NAFLD is mediated by TGs (31). This corroborates the conjecture based on our results. Significant differences in waist circumference and insulin resistance (IR) were found between the most severe phenotype (molecular subtype I) and the least severe phenotype (molecular subtype IV) (**Figures 4G, J** **)**. According to one study (32) with results consistent with our findings, the waist circumference is closely related to chronic inflammation and IR, and it might contribute to the development of NAFLD. Omega-6 (n-6) polyunsaturated fatty acids (PUFAs; e.g., arachidonic acid (AA)) and omega-3 (n-3) polyunsaturated fatty acids (e.g., eicosapentaenoic acid (EPA) and docosahexaenoic acid (DHA)) are precursors of lipid-mediated signaling molecules, and they play an important role in the regulation of inflammation. In general, eicosanes derived from n-6 PUFAs have pro-inflammatory effects, whereas eicosanes derived from n-3 PUFAs have anti-inflammatory effects (33). AA was relatively increased in molecular subtype IV (**Figure 4Q**), which represents the initial stage of NAFLD, indicating that AA increases at the initial stage of NAFLD and that it might be an early predictor of the disease, which is consistent with the results of a previous study (34). Both DHA and EPA can reduce hepatic lipid homeostasis damage, oxidative stress, and the inflammatory response (35) and inhibit the progression of NAFLD (36, 37). In our study, the DHA/EPA ratio in molecular subtypes II and III was lower than that in subtypes I and IV (**Figures 4R, S**). According to the immune score, the inflammatory response associated with molecular subtypes II and III was more severe than that with subtypes I and IV. Therefore, we speculate that DHA has a more inhibitory effect on the progression of NAFLD than EPA, which has been confirmed previously (38, 39).

## Conclusions

In this study, we present the following results: 1) we identified four molecular subtypes of NAFLD with unique pathogenic mechanisms and eigengenes; 2) we matched specific treatment strategies to specific disease-driving factors for specific patients; 3) we also evaluated the differences in drug efficacy among the four molecular subtypes. Moreover, these results could provide new insights into the underlying pathogenesis of and drug targets for NAFLD.

## Data Availability Statement

Publicly available datasets were analyzed in this study. This data can be found here: https://www.ncbi.nlm.nih.gov/geo/query/acc.cgi?acc=GSE48452; https://www.ncbi.nlm.nih.gov/geo/query/acc.cgi?acc=GSE89632; https://www.ncbi.nlm.nih.gov/geo/query/acc.cgi?acc=GSE151158.

## Author Contributions

ZL conceived the idea, performed all bioinformatics analysis, drew charts, and wrote the whole manuscript. YL and CY helped perform the analysis with constructive discussions and revised the manuscript. All authors contributed to the article and approved the submitted version.

## Funding

This work was supported by the Key Project of Developmental Biology and Breeding from Hunan Province (2022XKQ0205).

## Conflict of Interest

The authors declare that the research was conducted in the absence of any commercial or financial relationships that could be construed as a potential conflict of interest.

## Publisher’s Note

All claims expressed in this article are solely those of the authors and do not necessarily represent those of their affiliated organizations, or those of the publisher, the editors and the reviewers. Any product that may be evaluated in this article, or claim that may be made by its manufacturer, is not guaranteed or endorsed by the publisher.

## References

[1] Vilarinho S Ajmera V Zheng M Loomba R. Emerging Role of Genomic Analysis in Clinical Evaluation of Lean Individuals With Nafld. Hepatol (Baltimore Md) (2021) 74(4):2241–50. doi: 10.1002/hep.32047 PMC8463418 34233030

[2] Ahrens M Ammerpohl O von Schönfels W Kolarova J Bens S Itzel T. DNA Methylation Analysis in Nonalcoholic Fatty Liver Disease Suggests Distinct Disease-Specific and Remodeling Signatures After Bariatric Surgery. Cell Metab (2013) 18(2):296–302. doi: 10.1016/j.cmet.2013.07.004 23931760

[3] Arendt BM Comelli EM Ma DW Lou W Teterina A Kim T. Altered Hepatic Gene Expression in Nonalcoholic Fatty Liver Disease Is Associated With Lower Hepatic N-3 and N-6 Polyunsaturated Fatty Acids. Hepatol (Baltimore Md) (2015) 61(5):1565–78. doi: 10.1002/hep.27695 25581263

[4] Kriss M Golden-Mason L Kaplan J Mirshahi F Setiawan VW Sanyal AJ. Increased Hepatic and Circulating Chemokine and Osteopontin Expression Occurs Early in Human Nafld Development. PloS One (2020) 15(7):e0236353. doi: 10.1371/journal.pone.0236353 32730345 PMC7392333

[5] Clough E Barrett T. The Gene Expression Omnibus Database. Methods Mol Biol (Clifton NJ) (2016) 1418:93–110. doi: 10.1007/978-1-4939-3578-9_5 PMC4944384 27008011

[6] Leek JT Johnson WE Parker HS Jaffe AE Storey JD. The Sva Package for Removing Batch Effects and Other Unwanted Variation in High-Throughput Experiments. Bioinf (Oxford England) (2012) 28(6):882–3. doi: 10.1093/bioinformatics/bts034 PMC3307112 22257669

[7] Wilkerson MD Hayes DN. Consensusclusterplus: A Class Discovery Tool With Confidence Assessments and Item Tracking. Bioinf (Oxford England) (2010) 26(12):1572–3. doi: 10.1093/bioinformatics/btq170 PMC2881355 20427518

[8] Subramanian A Tamayo P Mootha VK Mukherjee S Ebert BL Gillette MA. Gene Set Enrichment Analysis: A Knowledge-Based Approach for Interpreting Genome-Wide Expression Profiles. Proc Natl Acad Sci USA (2005) 102(43):15545–50. doi: 10.1073/pnas.0506580102 PMC1239896 16199517

[9] Langfelder P Horvath S. Wgcna: An R Package for Weighted Correlation Network Analysis. BMC Bioinf (2008) 9:559. doi: 10.1186/1471-2105-9-559 PMC2631488 19114008

[10] Liberzon A Subramanian A Pinchback R Thorvaldsdóttir H Tamayo P Mesirov JP. Molecular Signatures Database (Msigdb) 3. Bioinf (Oxford England) (2011) 27(12):1739–40. doi: 10.1093/bioinformatics/btr260 PMC3106198 21546393

[11] Newman AM Liu CL Green MR Gentles AJ Feng W Xu Y. Robust Enumeration of Cell Subsets From Tissue Expression Profiles. Nat Methods (2015) 12(5):453–7. doi: 10.1038/nmeth.3337 PMC4739640 25822800

[12] Yoshihara K Shahmoradgoli M Martínez E Vegesna R Kim H Torres-Garcia W. Inferring Tumour Purity and Stromal and Immune Cell Admixture From Expression Data. Nat Commun (2013) 4:2612. doi: 10.1038/ncomms3612 24113773 PMC3826632

[13] Sutti S Albano E. Adaptive Immunity: An Emerging Player in the Progression of Nafld. Nat Rev Gastroenterol Hepatol (2020) 17(2):81–92. doi: 10.1038/s41575-019-0210-2 31605031 PMC7222953

[14] Al-Qarni R Iqbal M Al-Otaibi M Al-Saif F Alfadda AA Alkhalidi H. Validating Candidate Biomarkers for Different Stages of Non-Alcoholic Fatty Liver Disease. Medicine (2020) 99(36):e21463. doi: 10.1097/md.0000000000021463 32898995 PMC7478685

[15] Kozumi K Kodama T Murai H Sakane S Govaere O Cockell S. Transcriptomics Identify Thrombospondin-2 as a Biomarker for Nonalcoholic Steatohepatitis and Advanced Liver Fibrosis. Hepatol (Baltimore Md) (2021) 74(5):2452–66. doi: 10.1002/hep.31995 PMC8596693 34105780

[16] Krenkel O Tacke F. Macrophages in Nonalcoholic Fatty Liver Disease: A Role Model of Pathogenic Immunometabolism. Semin Liver Dis (2017) 37(3):189–97. doi: 10.1055/s-0037-1604480 28847030

[17] Friedman S Sanyal A Goodman Z Lefebvre E Gottwald M Fischer L. Efficacy and Safety Study of Cenicriviroc for the Treatment of Non-Alcoholic Steatohepatitis in Adult Subjects With Liver Fibrosis: Centaur Phase 2b Study Design. Contemp Clin Trials (2016) 47:356–65. doi: 10.1016/j.cct.2016.02.012 26944023

[18] Xi C Jia Z Xiaoli W Na Z He W Hao J. New Aspect of Liver Il-17(+)Γδ T Cells. Mol Immunol (2019) 107:41–3. doi: 10.1016/j.molimm.2018.12.030 30641412

[19] Sanyal AJ. Past, Present and Future Perspectives in Nonalcoholic Fatty Liver Disease. Nat Rev Gastroenterol Hepatol (2019) 16(6):377–86. doi: 10.1038/s41575-019-0144-8 31024089

[20] Yang M Khoukaz L Qi X Kimchi ET Staveley-O’Carroll KF Li G. Diet and Gut Microbiota Interaction-Derived Metabolites and Intrahepatic Immune Response in Nafld Development and Treatment. Biomedicines (2021) 9(12):1893. doi: 10.3390/biomedicines9121893 34944709 PMC8698669

[21] Willy JA Young SK Stevens JL Masuoka HC Wek RC. Chop Links Endoplasmic Reticulum Stress to Nf-Kb Activation in the Pathogenesis of Nonalcoholic Steatohepatitis. Mol Biol Cell (2015) 26(12):2190–204. doi: 10.1091/mbc.E15-01-0036 PMC4462938 25904325

[22] Henkel A Green RM. The Unfolded Protein Response in Fatty Liver Disease. Semin Liver Dis (2013) 33(4):321–9. doi: 10.1055/s-0033-1358522 PMC4110953 24222090

[23] Sumida Y Yoneda M. Current and Future Pharmacological Therapies for Nafld/Nash. J Gastroenterol (2018) 53(3):362–76. doi: 10.1007/s00535-017-1415-1 PMC5847174 29247356

[24] Barrow F Khan S Wang H Revelo XS. The Emerging Role of B Cells in the Pathogenesis of Nafld. Hepatol (Baltimore Md) (2021) 74(4):2277–86. doi: 10.1002/hep.31889 PMC8463421 33961302

[25] Habelhah H Frew IJ Laine A Janes PW Relaix F Sassoon D. Stress-Induced Decrease in Traf2 Stability Is Mediated by Siah2. EMBO J (2002) 21(21):5756–65. doi: 10.1093/emboj/cdf576 PMC131073 12411493

[26] Van Herck MA Weyler J Kwanten WJ Dirinck EL De Winter BY Francque SM. The Differential Roles of T Cells in Non-Alcoholic Fatty Liver Disease and Obesity. Front Immunol (2019) 10:82. doi: 10.3389/fimmu.2019.00082 30787925 PMC6372559

[27] Leiter LA Forst T Polidori D Balis DA Xie J Sha S. Effect of Canagliflozin on Liver Function Tests in Patients With Type 2 Diabetes. Diabetes Metab (2016) 42(1):25–32. doi: 10.1016/j.diabet.2015.10.003 26575250

[28] Seino Y Sasaki T Fukatsu A Ubukata M Sakai S Samukawa Y. Efficacy and Safety of Luseogliflozin as Monotherapy in Japanese Patients With Type 2 Diabetes Mellitus: A Randomized, Double-Blind, Placebo-Controlled, Phase 3 Study. Curr Med Res Opin (2014) 30(7):1245–55. doi: 10.1185/03007995.2014.912983 24708292

[29] Akuta N Watanabe C Kawamura Y Arase Y Saitoh S Fujiyama S. Effects of a Sodium-Glucose Cotransporter 2 Inhibitor in Nonalcoholic Fatty Liver Disease Complicated by Diabetes Mellitus: Preliminary Prospective Study Based on Serial Liver Biopsies. Hepatol Commun (2017) 1(1):46–52. doi: 10.1002/hep4.1019 29404432 PMC5747031

[30] Simon J Nuñez-García M Fernández-Tussy P Barbier-Torres L Fernández-Ramos D Gómez-Santos B. Targeting Hepatic Glutaminase 1 Ameliorates Non-Alcoholic Steatohepatitis by Restoring Very-Low-Density Lipoprotein Triglyceride Assembly. Cell Metab (2020) 31(3):605–22.e10. doi: 10.1016/j.cmet.2020.01.013 32084378 PMC7259377

[31] Xing J Guan X Zhang Q Chen S Wu S Sun X. Triglycerides Mediate Body Mass Index and Nonalcoholic Fatty Liver Disease: A Population-Based Study. Obes Facts (2021) 14(2):190–6. doi: 10.1159/000514848 PMC8138251 33780962

[32] Sheng G Xie Q Wang R Hu C Zhong M Zou Y. Waist-To-Height Ratio and Non-Alcoholic Fatty Liver Disease in Adults. BMC Gastroenterol (2021) 21(1):239. doi: 10.1186/s12876-021-01824-3 34034671 PMC8146664

[33] Patterson E Wall R Fitzgerald GF Ross RP Stanton C. Health Implications of High Dietary Omega-6 Polyunsaturated Fatty Acids. J Nutr Metab (2012) 2012:539426. doi: 10.1155/2012/539426 22570770 PMC3335257

[34] Sztolsztener K Chabowski A Harasim-Symbor E Bielawiec P Konstantynowicz-Nowicka K. Arachidonic Acid as an Early Indicator of Inflammation During Non-Alcoholic Fatty Liver Disease Development. Biomolecules (2020) 10(8):1133. doi: 10.3390/biom10081133 PMC7464179 32751983

[35] Liu L Hu Q Wu H Wang X Gao C Chen G. Dietary Dha/Epa Ratio Changes Fatty Acid Composition and Attenuates Diet-Induced Accumulation of Lipid in the Liver of Apoe(-/-) Mice. Oxid Med Cell Longevity (2018) 2018:6256802. doi: 10.1155/2018/6256802 PMC6261399 30538803

[36] Chen ZY Liu M Jing LP Xiao ML Dong HL Chen GD. Erythrocyte Membrane N-3 Polyunsaturated Fatty Acids Are Inversely Associated With the Presence and Progression of Nonalcoholic Fatty Liver Disease in Chinese Adults: A Prospective Study. Eur J Nutr (2020) 59(3):941–51. doi: 10.1007/s00394-019-01953-2 30937580

[37] Yang J Fernández-Galilea M Martínez-Fernández L González-Muniesa P Pérez-Chávez A Martínez JA. Oxidative Stress and Non-Alcoholic Fatty Liver Disease: Effects of Omega-3 Fatty Acid Supplementation. Nutrients (2019) 11(4):872. doi: 10.3390/nu11040872 PMC6521137 31003450

[38] Hong L Zahradka P Cordero-Monroy L Wright B Taylor CG. Dietary Docosahexaenoic Acid (Dha) and Eicosapentaenoic Acid (Epa) Operate by Different Mechanisms to Modulate Hepatic Steatosis and Hyperinsulemia in Fa/Fa Zucker Rats. Nutrients (2019) 11(4):917. doi: 10.3390/nu11040917 PMC6521162 31022865

[39] Kelley NS. Treatment of Nonalcoholic Fatty Liver Disease With Long-Chain N-3 Polyunsaturated Fatty Acids in Humans. Metab Syndrome Related Disord (2016) 14(9):417–30. doi: 10.1089/met.2016.0051 27710160

