# Supplementary material for: Identification of the Non-Alcoholic Fatty Liver Disease Molecular Subtypes Associated With Clinical and Immunological Features via Bioinformatics Methods
Source: Front Immunol. 2022 Jul 25;13:857892. doi: 10.3389/fimmu.2022.857892 (PMC9358963; doi:10.3389/fimmu.2022.857892)
Supplement: Supplementary file 1 [file Table_1.docx]

| **Supplemental Table 1. Collection of the clinical characteristics of NAFLD.** | |
| --- | --- |
| Datasets | Clinical characteristics |
| GSE48452 | steatosis (%) |
|  | lobular inflammation |
|  | ballooning |
|  | NAFLD activity score |
|  | fibrosis |
|  | body mass index (kg/m²) |
| GSE89632 | steatosis (%) |
|  | lobular inflammation |
|  | ballooning |
|  | NAFLD activity score |
|  | fibrosis |
|  | body mass index (kg/m²) |
|  | waist circumference (cm) |
|  | aspartate transaminase (u/l) |
|  | alanine transaminase (u/l) |
|  | alkaline phosphatase (u/l) |
|  | triglycerides (mmol/l) |
|  | total cholesterol (mmol/l) |
|  | ldl cholesterol (mmol/l) |
|  | hdl cholesterol (mmol/l) |
|  | fasting glucose (mmol/l) |
|  | homa-insulin resistance |
|  | hemoglobin a1c |
|  | liver arachidonic acid (% of total lipids) |
|  | liver eicosapentaenoic acid (% of total lipids) |
|  | liver docosahexaenoic acid (% of total lipids) |
| GSE151158 | steatosis (%) |
|  | lobular inflammation |
|  | ballooning |
|  | NAFLD activity score |
|  | aspartate transaminase (u/l) |
|  | alanine transaminase (u/l) |
|  | alkaline phosphatase (u/l) |
| NAFLD: non-alcoholic fatty liver disease; LDL: low density lipoprotein; HDL: high density lipoprotein | |
