# Supplementary material for: Identification of the Non-Alcoholic Fatty Liver Disease Molecular Subtypes Associated With Clinical and Immunological Features via Bioinformatics Methods
Source: Front Immunol. 2022 Jul 25;13:857892. doi: 10.3389/fimmu.2022.857892 (PMC9358963; doi:10.3389/fimmu.2022.857892)
Supplement: Supplementary file 2 [file Table_2.docx]

| **Supplemental Table 2.** **Summary of the relationships among Molecular subtypes, Gene modules, Central-eigengenes, Biological processes, Molecular functions and Cellular components.** | | | | | | |
| --- | --- | --- | --- | --- | --- | --- |
| Molecular subtype  (three classification method) | Module  (eight classification method) | The most connected genes (Central-eigengenes) and Connective frequency of each gene | | Biological processes | Molecular functions | Cellular components |
| Ⅰ | turquoise | STAT3  C3  MAPK1  FN1  APP  IL13  IL17A  TP53  IL2  STAT5B  CSF2  ITGB1 | 19  15  15  13  12  12  12  12  11  11  10  10 | *regulation of cellular response to tunicamycin  *regulation of cellular response to thapsigargin  *positive regulation of memory T cell differentiation  *positive regulation of CD8-positive, alpha-beta T cell proliferation  *regulation of activation of membrane attack complex  *positive regulation of T-helper 17 cell lineage commitment  *positive regulation of amyloid fibril formation  *activation of cysteine-type endopeptidase activity involved in apoptotic signaling pathway  *positive regulation of NK T cell proliferation  *T-helper 1 cell cytokine production  *calcium-independent cell-matrix adhesion | *interleukin-12 binding  *interleukin-12 receptor binding  *interleukin-2 receptor binding  *TFIID-class transcription factor complex binding  *glucocorticoid receptor binding | *interleukin-23 complex  *interleukin-12 complex  *nuclear envelope lumen  *late endosome lumen |
| Ⅱ | brown | STAT1  HLA-DRA  VCAM1  TNFRSF1B  CTSS  FYN  GBP1  IRF8  TLR4  CXCL12  PSMB9  CD4  CD53 | 9  7  7  5  4  4  4  4  4  3  3  2  2 | \| *peptide antigen assembly with MHC class II protein complex \| \| --- \| \| *detection of lipopolysaccharide \| \| *regulation of T cell activation via T cell receptor contact with antigen bound to MHC molecule on antigen presenting cell \| \| *negative regulation of MyD88-independent toll-like receptor signaling pathway \| \| *interleukin-21-mediated signaling pathway \| \| *detection of mechanical stimulus involved in sensory perception of pain \| \| *amyloid-beta clearance \| \| *complement activation, alternative pathway \| \| *interleukin-15-mediated signaling pathway \| | \| *lipopolysaccharide receptor activity \| \| --- \| \| *pattern recognition receptor activity \| \| *tumor necrosis factor receptor binding \| \| *MHC protein binding \| | \| *lipopolysaccharide receptor complex \| \| --- \| \| *MHC class II protein complex \| \| *immunological synapse \| \| *tertiary granule lumen \| |
|  | green | \| IL10 \| \| --- \| \| CCR7 \| \| CD274 \| \| ICAM1 \| \| IL18RAP \| \| IRF1 \| \| JAK3 \| \| NOD2 \| | \| 4 \| \| --- \| \| 3 \| \| 3 \| \| 3 \| \| 2 \| \| 2 \| \| 2 \| \| 2 \| | \| *neutrophil aggregation \| \| --- \| \| *regulation of dendritic cell cytokine production \| \| *sequestering of zinc ion \| \| *negative regulation of CD8-positive, alpha-beta T cell activation \| \| *peptidyl-cysteine S-nitrosylation \| \| *regulation of CD8-positive, alpha-beta T cell differentiation \| \| *positive regulation of tolerance induction \| \| *positive regulation of dendritic cell antigen processing and presentation \| \| *negative regulation of T-helper 1 type immune response \| \| *cytoplasmic sequestering of NF-kappaB \| \| *nucleotide-binding oligomerization domain containing 2 signaling pathway \| \| *regulation of CD8-positive, alpha-beta T cell activation \| \| *negative regulation of interleukin-12 production \| \| *regulation of interleukin-10 secretion \| | \| *Toll-like receptor 4 binding \| \| --- \| \| *arachidonic acid binding \| \| *Toll-like receptor binding \| \| *RAGE receptor binding \| \| *chemokine binding \| \| *tumor necrosis factor-activated receptor activity \| | \| *tertiary granule membrane \| \| --- \| \| *external side of plasma membrane \| \| *extracellular exosome \| \| *specific granule membrane \| \| *plasma membrane raft \| |
|  | pink | \| CD86 \| \| --- \| \| ITGB2 \| \| CSF1R \| \| FCER1G \| \| LILRB2 \| \| TLR2 \| \| CD163 \| \| C1QB \| \| CD80 \| \| LCP2 \| | \| 11 \| \| --- \| \| 6 \| \| 6 \| \| 5 \| \| 5 \| \| 4 \| \| 4 \| \| 4 \| \| 4 \| \| 4 \| | \| *positive regulation of type III hypersensitivity \| \| --- \| \| *positive regulation of type I hypersensitivity \| \| *Fc receptor mediated inhibitory signaling pathway \| \| *regulation of dendritic cell differentiation \| \| *positive regulation of interleukin-2 biosynthetic process \| \| *positive regulation of regulatory T cell differentiation \| \| *production of molecular mediator involved in inflammatory response \| \| *mast cell activation \| \| *positive regulation of myeloid leukocyte mediated immunity \| | \| *inhibitory MHC class I receptor activity \| \| --- \| \| *complement component C3b binding \| \| *protein phosphatase 1 binding \| \| *MHC class I protein binding \| \| *immunoglobulin binding \| \| *amyloid-beta binding \| \| *coreceptor activity \| \| *virus receptor activity \| | \| *ficolin-1-rich granule membrane \| \| --- \| \| *protein complex involved in cell adhesion \| \| *tertiary granule membrane \| |
| **Ⅲ** | black | IL4  STAT5A  TRAF2  CD19  IL23R  IFNB1  IL5  TLR9  TNFRSF13C  TRAF5 | 7  7  6  6  5  5  4  4  4  4 | \| *negative regulation of interleukin-13 secretion \| \| --- \| \| *negative regulation of interleukin-13 production \| \| *tolerance induction to self antigen \| \| *negative regulation of T-helper 2 cell cytokine production \| \| *leukotriene signaling pathway \| \| *neutrophil clearance \| \| *tumor necrosis factor superfamily cytokine production \| \| *tumor necrosis factor production \| \| *negative regulation of type 2 immune response \| \| *negative regulation of interleukin-5 production \| \| *regulation of MHC class I biosynthetic process \| \| *regulation of toll-like *receptor 9 signaling pathway \| \| *regulation of memory T cell differentiation \| \| *germ cell migration \| \| *coronary artery morphogenesis \| \| *regulation of extracellular matrix assembly \| | \| *leukotriene B4 receptor activity \| \| --- \| \| *type II transforming growth factor beta receptor binding \| \| *thioesterase binding \| \| *tumor necrosis factor receptor binding \| \| *type I interferon receptor binding \| | \| *CD40 receptor complex \| \| --- \| \| *plasma membrane *receptor complex \| \| *membrane raft \| \| *plasma membrane protein complex \| \|  \| |
| **Ⅳ** | blue | \| CD28 \| \| --- \| \| CCR5 \| \| CD247 \| \| IL15 \| \| CCL18 \| \| CXCR3 \| \| EOMES \| \| HRAS \| \| PDCD1 \| \| SH2D1A \| \| TNFRSF4 \| | \| 5 \| \| --- \| \| 3 \| \| 3 \| \| 3 \| \| 2 \| \| 2 \| \| 2 \| \| 2 \| \| 2 \| \| 2 \| \| 2 \| | \| *positive regulation of interleukin-17 production \| \| --- \| \| *regulation of interleukin-6 biosynthetic process \| \| *cytokine metabolic process \| \| *lymph node development \| \| *positive regulation of natural killer cell mediated cytotoxicity \| \| *cellular defense response \| \| *regulation of T cell apoptotic process \| \| *positive regulation of interleukin-4 production \| \| *T cell selection \| \| *alpha-beta T cell differentiation involved in immune response \| \| *cellular response to gamma radiation \| | \| *chemokine binding \| \| --- \| \| *tumor necrosis factor-activated receptor activity \| \| *chemokine receptor activity \| \| *coreceptor activity \| \| *cytokine binding \| | \| *immunological synapse \| \| --- \| \| *external side of plasma membrane \| \| *plasma membrane receptor complex \| |
|  | grey | TNF  ITGAM  FOXP3  LCK  TRAF6  TBX21  CD5  CCRL2  CXCL10  IL7R  PPBP  THY1 | 12  10  8  7  7  6  5  4  4  4  4  4 | \| *B cell apoptotic process \| \| --- \| \| *negative regulation of T cell cytokine production \| \| *positive thymic T cell selection \| \| *positive regulation of interleukin-8 biosynthetic process \| \| *chronic inflammatory response \| \| *regulation of chronic inflammatory response \| \| *negative regulation of collagen biosynthetic process \| \| *positive regulation of ceramide biosynthetic process \| \| *negative regulation of T-helper 17 cell differentiation \| \| *regulation of T cell chemotaxis \| \| *positive regulation of T cell differentiation in thymus \| \| *CD4-positive, alpha-beta T cell lineage commitment \| | \| *CXCR3 chemokine receptor binding \| \| --- \| \| *thioesterase binding \| \| *CD4 receptor binding \| \| *CXCR chemokine receptor binding \| \| *protein kinase B binding \| \| *opsonin binding \| \| *FK506 binding \| \| *CARD domain binding \| \| *tumor necrosis factor receptor binding \| \| *complement binding \| | \| *phagocytic vesicle lumen \| \| --- \| \| *CD40 receptor complex \| \| *T cell receptor complex \| \| *immunological synapse \| \| *integrin complex \| \| tertiary granule lumen \| \| *plasma membrane receptor complex \| \| *external side of plasma membrane \| |
|  | | | | | | |
